# Neuroprotective Effect of Resveratrol via Activation of Sirt1 Signaling in a Rat Model of Combined Diabetes and Alzheimer’s Disease

**DOI:** 10.3389/fnins.2019.01400

**Published:** 2020-01-21

**Authors:** XingRong Ma, ZhiKun Sun, Xiao Han, Shujian Li, Xiaofeng Jiang, Shuai Chen, Jiewen Zhang, Hong Lu

**Affiliations:** ^1^Department of Neurology, The First Affiliated Hospital, Zhengzhou University, Zhengzhou, China; ^2^Department of Neurology, Henan Provincial People’s Hospital, Zhengzhou, China

**Keywords:** resveratrol, Alzheimer’s disease, diabetes mellitus, oxidative stress, Aβ1-40, neuroprotective, Sirt1, resteratrol

## Abstract

**Background:**

Alzheimer’s disease (AD) and diabetes mellitus (DM) often coexist in patients because having one of these conditions increases risk for the other. These two diseases share several pathophysiological mechanisms, such as specific inflammatory signaling pathways, oxidative stress, and cell apoptosis. It is still unclear exactly which mechanisms associated with DM are responsible for increased AD risk. Studies have found that even transient elevation of brain Aβ levels can allow T2DM to slightly disrupt the neural milieu in a way that encourages pathologies associated with the onset of memory deficits and AD. A recent study argues that a potential common pathogenetic mechanism underlying both DM and AD is evidenced by the cooccurrence of amyloid brain legions and deposits containing both tau and Aβ in pancreatic β cells. Given these links, an investigation detailing disease mechanisms as well as treatment options for patients with cooccurring DM and AD is urgently needed. The biological effects of resveratrol relevant to DM and AD treatment include its abilities to modulate oxidative stress and reduce inflammation. A rat model of DM and concomitant AD was created for this study using intraperitoneal injection of streptozotocin and hippocampal injection of Aβ1–40 to characterize resveratrol’s potential protective action.

**Results:**

Resveratrol significantly increased the Sirt1 expression, inhibited the memory impairment, the increased acetylcholinesterase, malondialdehyde, interleukin-1β and interleukin 6 levels, and the decreased levels of choline acetyltransferase (ChAT), superoxide dismutase (SOD), and glutathione in this rat model of diabetes and concomitant AD. The Sirt 1 inhibitor EX527 partially reversed the effects of resveratrol.

**Conclusion:**

This study suggests that resveratrol may have a neuroprotective action through activation of Sirt1 signaling in diabetes and AD with concurrent onset.

## Introduction

Alzheimer’s disease (AD) is a disorder involving selective central nervous system degeneration, including neuron loss and gradual development of amyloid plaques and neurofibrillary tangles (Iwatsubo, 2000). At present, the cause of AD remains unclear, although aberrant Aβ production or clearance from the brain is currently considered the most probable cause (Pereira et al., 2004). The deleterious effect of Aβ involves oxidative stress (Butterfield et al., 2013), inflammatory responses, neuronal apoptosis, etc. (Agostinho et al., 2010). Owing to the recent rapid growth of older populations, the incidence of AD has naturally increased, imposing a substantial cost on the economy and impacting the well-being of families and society at large. The 2019 Alzheimer’s Association Report states that Americans currently living with AD number about 5.8 million, and by 2050, the prevalence of AD will reach more than 13.8 million cases in the United States alone. In 2019, an estimated $290 billion will be spent providing health services, long-term care including assistance with daily living, and hospice care to dementia patients aged 65 or older (Alzheimer’s Association, 2019).

Diabetes mellitus (DM) is a disease of disturbed metabolism wherein aberrant insulin secretion and/or action leads to hyperglycemia (Marklova, 2001). Modern improvements in living coupled with decreased activity levels have increased the prevalence of both obesity and DM. A retrospective cohort study reported an increase in the rate of type 2 diabetes mellitus (T2DM) from 2.39% in 2000 to 5.32% in 2013 (Sharma et al., 2016). Approximately 415 million people worldwide had DM in 2015 with 90% of the cases being T2DM (Shi and Hu, 2014). In 2017, approximately 451 million people worldwide between the ages of 18 and 99 years suffered from diabetes, and by 2045, prevalence will climb to approximately 693 million cases. In 2017 alone, approximately USD 850 billion was spent worldwide on diabetes care (Cho et al., 2018).

Both AD and DM are associated with aging, and because onset of either disease increases risk for the other, concurrent onset is common. A longitudinal population-based study has revealed high risk for AD in DM patients (Biessels et al., 2005). Dementia is two to three times more common in patients with DM than in patients without DM (Sridhar et al., 2015). However, it is still unclear exactly which mechanisms associated with DM are responsible for increased AD risk. Su et al. (2019) found that even transient elevation of brain Aβ levels can allow T2DM to slightly disrupt the neural milieu in a way that encourages pathologies associated with the onset of memory deficits and AD. Martinez-Valbuena et al. (2019) argue that a potential common pathogenetic mechanism underlying both DM and AD is evidenced by the co-occurrence of amyloid brain lesions and deposits containing both tau and Aβ in pancreatic β cells. Therefore, an investigation detailing disease mechanisms as well as treatment options for patients with co-occurring DM and AD is urgently needed.

The natural phenol resveratrol occurs in several common foods, most notably in grapes. Research has cataloged its antioxidant, anti-inflammatory (Wood et al., 2010), and anti-carcinogenic effects (Athar et al., 2009). Sirt1, a homolog of Sirt2, is a highly conserved NAD+-dependent deacetylase. Sirt1 operates via histone/non-histone deacetylase activity and helps to regulate many cell processes, such as DNA damage, apoptosis, transcription, and metabolism through a reversible acetylation–deacetylation reaction (Paraiso et al., 2013). In addition, Sirt1 is also involved in caloric restriction and aging (Tissenbaum and Guarente, 2001). Studies have suggested that resveratrol is a Sirt1 activator (Pallas et al., 2009; Wu et al., 2011). However, studies evaluating the effect of resveratrol on the pathophysiology of co-occurring DM and AD are still lacking. In this study, we used intraperitoneal injections of streptozotocin with hippocampal injections of Aβ1–40 to model diabetes with co-occurring AD in rats to examine resveratrol’s potential to alter the pathophysiology of DM and AD.

## Materials and Methods

### Reagents

Aβ1–40 (Sigma-Aldrich, St Louis, MO, United States) was dissolved (10 μg/μl) in sterile saline solution at 37°C for at least 7 days. After dissolution of the Sirt1 inhibitor EX527 (Tocris Bioscience, Bristol, United Kingdom) in dimethyl sulfoxide (DMSO), the resultant mixture was diluted with saline to reach the appropriate concentration (final DMSO concentration <2%). Streptozotocin (STZ) (Sigma-Aldrich, St. Louis, MO, United States) was dissolved in 0.1 M citrate buffer (pH 4.5). Resveratrol (Sigma-Aldrich, St. Louis, MO, United States) was dissolved in sterile saline. Nanjing Jiancheng Bioengineering Institute (Nanjing, China) supplied the kits for measuring AchE and ChAT activity. Thermo Fisher Scientific (Shanghai, China) supplied the MDA and SOD measurement kits. Cayman Chemical (Ann Arbor, MI, United States) supplied the GSH assay kit. Nanjing KeyGEN Biotech. Co., Ltd. (Nanjing, China), supplied the interleukin (IL)-1β and IL-6 enzyme-linked immunosorbent assay (ELISA) kits. Sigma-Aldrich (Beijing, China) supplied the mouse monoclonal anti-Sirt1 antibody and the mouse monoclonal antibeta-actin antibody. Pierce Biotechnology (Rockford, IL, United States) supplied the bicinchoninic acid (BCA) assay. Amersham (United Kingdom) supplied the ECL advance Western blotting detection kit. All kits were purchased.

### Animals

Wistar rats (Henan Laboratory Animal Research Center, Zhengzhou, China) of approximately 8–10 months of age (250–300 g) were used in this study and were held in conventional cages with *ad libitum* feeding, a constant ambient temperature of 22 ± 2°C, humidity of 55 ± 5%, and a light–dark cycle of 12 h (7:00–19:00). All aspects of this research have complied with the Guideline on the Humane Treatment of Laboratory Animals instituted by the Ministry of Science and Technology of the People’s Republic of China. The Committee on Ethics in Life Sciences of Zhengzhou University approved this study.

### Experimental Design

**FIGURE 1:**
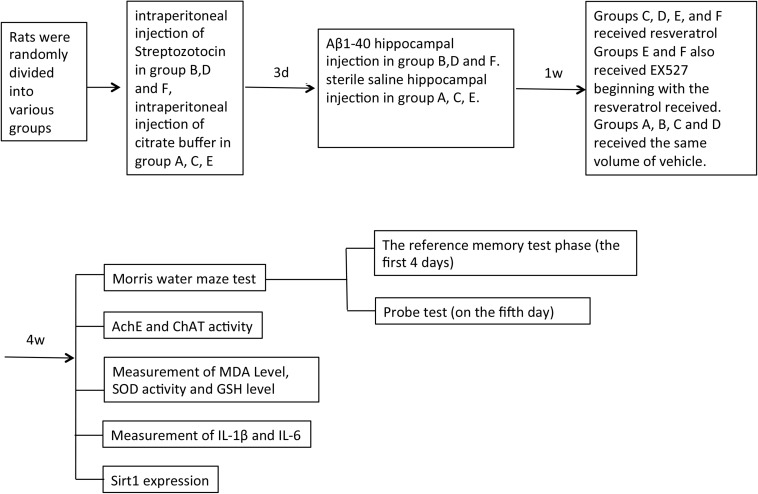
Experiment flow chart.

Groups (*n* = 21) were formed by random assignment of rats as follows: normal control group to receive sham operation (group A), group treated to establish a concurrent diabetes and AD disease model (group B), resveratrol control group to receive sham operation with resveratrol (group C), model rats receiving treatment with resveratrol (group D), resveratrol and EX527 (Sirt1 inhibitor) control group to receive sham operation with resveratrol and EX527 (group E), and model rats receiving treatment with both resveratrol and EX527 (group F). The concurrent diabetes and AD disease model was established by intraperitoneal injection of streptozotocin and subsequent hippocampal injection of Aβ1–40 in groups B, D, and F only. Only citrate buffer and sterile saline with neither streptozotocin nor Aβ1–40 were injected in groups A, C, and E. Resveratrol (25 mg/kg) was orally administered to groups C–F daily from 1 to 5 weeks postoperation. One 5 mg/kg dose of EX527 was also administered to groups E and F through intraperitoneal injection every 2 days (beginning concurrently with the first resveratrol dose) for a total of four doses. An equivalent volume of vehicle was administered to groups A–D (Figure 1).

### Diabetes and Alzheimer’s Disease Rat Model

A single 55 mg/kg dose of dissolved streptozotocin (in 0.1 M citrate buffer, pH 4.5) administered via intraperitoneal injection was used to induce experimental DM in rats. Three days postinjection, blood samples were collected from fasting rats (12 h overnight) by tail vein sampling, and a glucose meter with test strips (Ascensia Entrust, Bayer Polychem Ltd., Thane, India) was used to determine blood glucose level. A fasting blood glucose level over 16.7 mmol/dl was taken to indicate diabetes, and only rats exceeding the cutoff were retained for subsequent experiments. Ten percent chloral hydrate (0.4 ml/100 g; Sigma-Aldrich, St. Louis, MO, United States) administered via intraperitoneal injection was used to anesthetize diabetic rats. Atropine sulfate (0.1 mg/kg, i.m., Polfa Warszawa, Poland) was also given to avoid intraoperative respiratory depression and distress in rats. Stereotaxic surgical technique including a frame (Stoelting Co., United States) was used to localize the hippocampus of anesthetized rats. After scalp incision, a mini-drill was used to drill through the cranium to a depth of 2.8 mm. The CA1 region of the hippocampus was localized 2.0 mm lateral and −3.0 mm anterior to the posterior fontanel, in accordance with the Paxinos and Watson rat atlas. One microliter of Aβ1–40 was gradually injected over a 10-min period bilaterally into each hippocampus using a 26-gauge needle connected to a microsyringe. Following injection, the needle was slowly removed. Identical surgical procedure was followed for control rats, with the exception of injection of 1 μl of sterile saline instead of Aβ1–40.

### Morris Water Maze

The MWM (Chinese Academy of Medical Sciences) was used to assess spatial learning and memory function at 5 weeks postoperation as has been described in previous studies (Ma et al., 2013; Sun et al., 2014). The design of the maze included a painted circular pool (120 cm diameter; 30 cm depth) with a hidden platform (9 cm diameter) resting obscured 1.5 cm below the surface. For the test, rats were first trained to seek the submerged platform and exit the pool. Titanium dioxide was used to opacify the water in the pool, and during all training and testing procedures, water temperature was kept at 22°C. The pool was partitioned into four sections: north (target), south (opposite), east (adjacent 1), and west (adjacent 2). A tracking system connected to a computer and equipped with a camera was used to record experiments.

The MWM test was continually performed for 5 days. During the first test phase, rats were trained to develop a reference memory. The first phase required 4 days, during which rats completed four trials per day with a 30- to 40-min interval between trials for a total of 16 trials in the entire phase. Each trial began with the rat being placed in one randomly selected section of the maze facing the wall. Each rat was given 180 s to find and climb atop the hidden platform. The rat was removed 30 s after successfully mounting the platform and placed in a warmed holding cage. If a rat was unsuccessful in finding and climbing the submerged platform within 180 s, then it was shown the path to the platform by the operator’s gentle guidance and was left on the platform for a period of 30 s before removal and transfer to the warmed holding cage. The rats’ movements were tracked by a camera mounted above the pool. The time necessary for rats to locate and climb the platform (escape latency) as well as the distance swum (escape distance) were determined using MWM software (Shanghai Jiliang Software Technology Co., Ltd., China) calculations based upon video review. The second phase of testing beginning on the fifth day included a probe test to examine memory maintenance or deficit after platform removal. Rats were initially positioned in one of the sections beside the platform (adjacent 1 or adjacent 2 quadrant) and animals were tracked during a free swimming period of 120 s. Recordings were taken to note time and distance spent in the target quadrant, expressed as a percentage of total time and distance swum. To ensure proper blinding procedure, the probe test was conducted by an administrator with no knowledge of the experimental design.

### Sample Preparation and Biochemical Evaluations

Samples were prepared as has been described in prior studies (Ma et al., 2013). Administration of a pentobarbital overdose was used to euthanize rats. After immediate removal of the brains, some (*n* = 6) were stored at −80°C for future Western blot testing and others (*n* = 15) were immediately processed by isolating the cerebral cortex and hippocampus on ice. The isolated cortical and hippocampal tissue was homogenized in 0.1 M phosphate-buffered saline (pH 7.4) and spun down at 10,000 × *g* at 4°C for 15 min in a centrifuge to separate the supernatant and debris. Supernatant protein concentration was quantified via the BCA Protein assay kit (Pierce Biotechnology, Rockford, IL, United States) with BSA used as a standard.

### AchE and ChAT Activity

AchE activity was assessed via Ellman’s reagent colorimetric assay as has been described in prior papers (Ellman et al., 1961). In brief, 0.75 mM acetylthiocholine and 0.5 mM 5,5-dithiobis(2-nitrobenzoic acid) (DTNB) in 5 mM HEPES buffer (pH 7.5) was used to measure AchE activity at 412 nm. ChAT activity was assessed via the radiochemical method as has been described in previous studies (Sterri and Fonnum, 1980). Following incubation of samples with ^14^C-labeled acetyl coenzyme A, the reaction was interrupted to measure the resultant quantity of ^14^C-labeled acetylcholine at 324 nm.

### Measurement of Levels of MDA and GSH and SOD Activity

Malondialdehyde level was determined using thiobarbituric acid spectrophotometric colorimetry as has been previously reported (Ma et al., 2013). In brief, MDA, the degraded product of peroxidized lipids, was condensed with thiobarbituric acid resulting in the formation of a red product that displayed a maximum absorption peak at 532 nm, which was measured using the SP-75 ultraviolet spectrophotometer (Shanghai Spectrum, Shanghai, China). Activity of SOD was determined via an assay based upon SOD’s ability to inhibit the photoreduction of nitroblue tetrazolium (NBT) by the superoxide anion radical resulting from the reaction of xanthine and xanthine oxidase. SOD was measured using nitrite spectrophotometry as has been previously reported (Ma et al., 2013). The enzymatic recycling method based on DTNB and glutathione reductase was utilized to quantify glutathione (GSH) level spectrophotometrically, as has been previously reported (Ma et al., 2013).

### Quantification of IL-1β and IL-6

IL-1β and IL-6 levels were detected using ELISA kits following the manufacture’s protocol. A SpectraMax M2 spectrometer (Molecular Devices, Sunnyvale, CA, United States) was used to analyze resultant data.

### Western Blot

Previously stored brain tissue was homogenized for 30 min using lysis buffer [10 mM Tris, pH 7.4, 100 mM NaCl, 1 mM EDTA, 1 mM EGTA, 1 mM NaF, 20 mM Na_4_P_2_O_7_, 2 mM Na_3_VO_4_, 0.1% sodium dodecyl sulfate, 0.5% sodium deoxycholate, 1% Triton-X 100, 10% glycerol, 1 mM phenylmethylsulfonyl fluoride (made from a 0.3 M stock in DMSO), 60 μg/ml aprotinin, 10 μg/ml leupeptin, 1 μg/ml pepstatin]. The sample was then centrifuged for 20 min at 2,500 × *g* at a temperature of 4°C to retrieve the supernatant. The BCA assay (Pierce Biotechnology, Rockford, IL, United States) was used to determine the total concentration of protein in the sample. Western blot testing was performed according to previously described procedure (Sun et al., 2008; Lee et al., 2013). Protein samples (20 μg each) were boiled (100°C) in buffer (Fermentas) for 10 min. Sodium dodecyl sulfate polyacrylamide gel (8–10%) was used to separate the proteins, which were then moved to polyvinylidene difluoride (PVDF) membranes (Bio-Rad, Hercules, CA, United States) via electrotransfer. A solution of 5% non-fat milk in TBST buffer (10 mM Tris–HCl, pH 8.0, 150 mM NaCl, and 0.2% Tween-20) was used to block the membranes by soaking for 1 h at room temperature. Next, membranes were incubated overnight at 4°C with one of two primary antibodies: either a 1:1,000 dilution of Sirt1 antibody (Sigma-Aldrich, Beijing, China) or a 1:5,000 dilution of beta-actin antibody (Sigma-Aldrich, Beijing, China). Following primary incubation, membranes were thoroughly washed two times with TBST and then incubated for 1 h at room temperature with horse horseradish peroxidase secondary antibodies (1:5000 dilution of anti-mouse/rabbit horse horseradish peroxidase). Amersham ECL Advance Western Blotting Detection kit (Amersham, United Kingdom) was used for signal development. Finally, an Axiocam digital microscope camera (ZEISS, Germany) and KS400 image analysis system software (Version 3.0) were used to quantify band intensities via densitometry.

### Statistical Analysis

Each test and assay was run in duplicate in three to five separate experimental trials. One-way ANOVA and a two-tailed *t*-test were run using SPSS Statistics 16.0 software (SPSS, Chicago, IL, United States), with *P* < 0.05 being statistically significant. Data are expressed as mean ±standard deviation.

## Results

### Effects of Resveratrol on Memory Deficits in Rat Model of Diabetes and Alzheimer’s Disease

**FIGURE 2:**
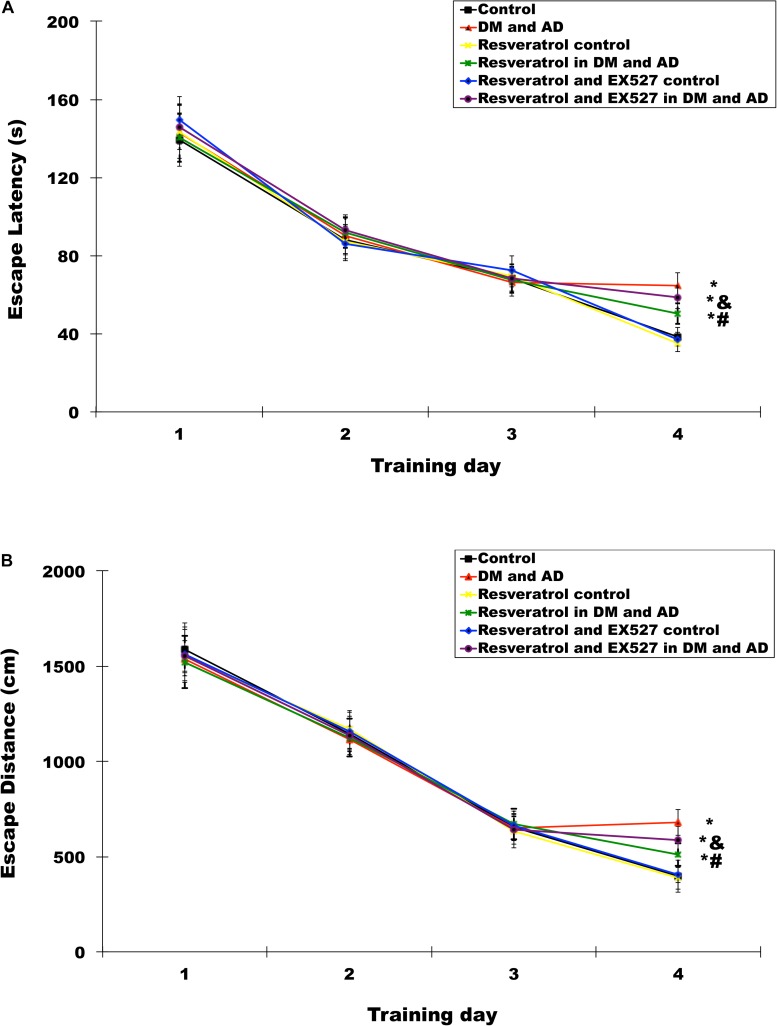
Effects of resveratrol on memory impairment in Diabetes mellitus (DM) and Alzheimer’s disease (AD) rats. The Morris water maze (MWM) was used to test rats’ memories by measuring escape latency **(A)** and escape distances **(B)**. Three independent experiments were performed. Data are expressed as mean ±SD; ^∗^*P* < 0.05 vs. control group, ^#^*P* < 0.05 vs. DM and AD group, ^&^*P* < 0.05 vs. resveratrol in DM and AD group.

The time needed for rats to find and mount a water maze platform (escape latency) and total swimming distance before escape (escape distance) were measured using the MWM test. For the first 3 days, there was no significant difference in escape latency (Figure 2A) (*P* > 0.05) or distance (Figure 2B) (*P* > 0.05) for each group. However, by the fourth day, the model group exhibited significantly longer escape latency and escape distance compared to controls (*P* < 0.05). Furthermore, escape latency and distance were significantly shorter in the rats receiving treatment with resveratrol versus model rats (*P* < 0.05) (Figure 2). However, escape latency and escape distance in rats treated with both resveratrol and EX527 were significantly longer compared to rats treated with resveratrol alone (*P* < 0.05) (Figure 2), indicating that the Sirt1 inhibitor EX527 can partially reverse the effects of resveratrol.

**FIGURE 3:**
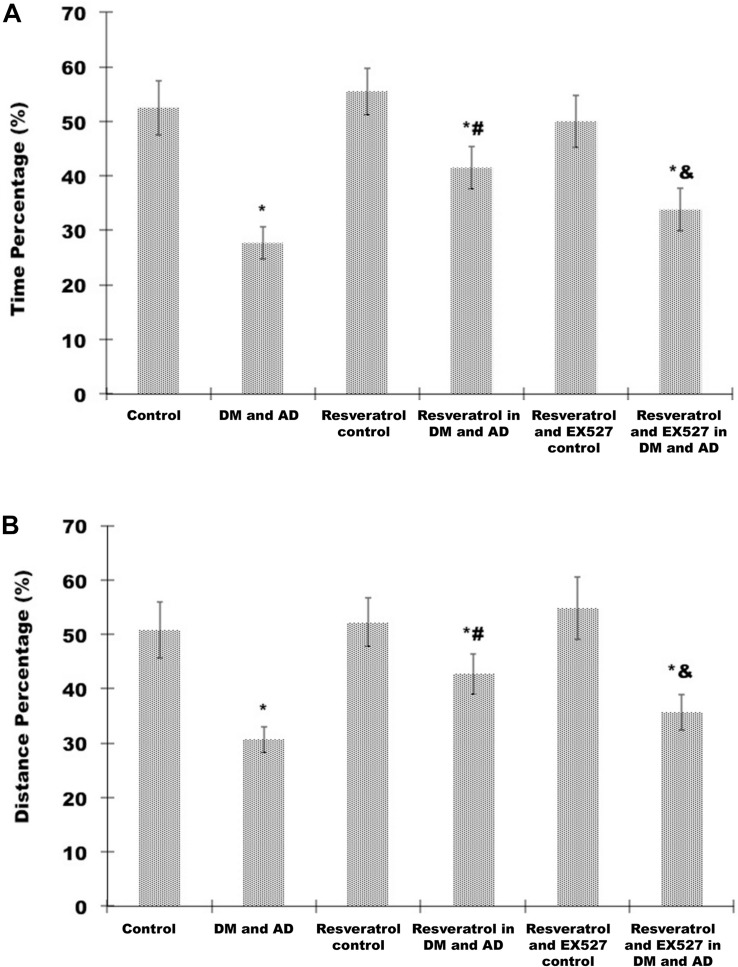
Effects of resveratrol on memory impairment in DM and AD rats. A probe test was used to analyze maintenance of memory in the MWM by measuring the percentage of time spent **(A)** and distance swum **(B)** in the target quadrant. Three independent experiments were performed. Data are expressed as mean ±SD. ^∗^*P* < 0.05 vs. control group, ^#^*P* < 0.05 vs. DM and AD group, ^&^*P* < 0.05 vs. resveratrol in DM and AD group.

Probe testing showed that swim time (Figure 3A) and distance swum (Figure 3B) in the target quadrant (expressed as a percentage of total time and distance swum) were significantly shorter in the model group than in the control group (*P* < 0.05). Compared with model rats, percentage of time (Figure 3A) and percentage of distance swum (Figure 3B) were significantly greater in rats treated with resveratrol (*P* < 0.05) (Figure 3). However, the percentage of time (Figure 3A) and percentage of distance swum (Figure 3B) in rats receiving both resveratrol and EX527 were both significantly less compared to rats receiving only resveratrol (*P*<0.05) (Figure 3), indicating the ability of the Sirt1 inhibitor EX527 to partially reverse the effects of resveratrol.

### Cortical and Hippocampal Activity of Acetylcholinesterase and Choline Acetyltransferase

As shown in Table 1, cortical and hippocampal AchE activity was significantly increased, while ChAT activity was significantly decreased in the brains of rats from the disease model group versus healthy controls (*P* < 0.05). In contrast, a significant decrease in AchE activity and simultaneous significant increase in ChAT activity were observed in the cortex and hippocampus of rats receiving resveratrol treatment versus model rats (*P* < 0.05) (Table 1). However, cortical ad hippocampal AchE activity was significantly increased, and ChAT activity was significantly decreased (*P* < 0.05) in the brains of resveratrol- and EX527-treated rats versus the resveratrol-only group (*P* < 0.05) (Table 1), indicating the ability of the Sirt1 inhibitor EX527 to partially reverse the effects of resveratrol.

**TABLE 1:** The AchE and ChAT activity in the cortex and hippocampus.

	AchE (U/mg protein)		ChAT (U/mg protein)	
	Cortex	Hippocampus	Cortex	Hippocampus
Control	1.83 ± 0.15	2.76 ± 0.21	365.46 ± 33.46	413.26 ± 37.28
DM and AD	3.96 ± 0.25^∗^	4.77 ± 0.35^∗^	238.13 ± 19.37^∗^	273.35 ± 23.27^∗^
Resveratrol control	1.79 ± 0.28	2.69 ± 0.30	374.67 ± 24.58	421.47 ± 27.69
Resveratrol in DM and AD	2.64 ± 0.22^#^	3.23 ± 0.31^#^	312.36 ± 25.29^#^	347.17 ± 28.71^#^
Resveratrol and EX527 control	1.85 ± 0.19	2.80 ± 0.24	379.37 ± 27.41	418.35 ± 31.79
Resveratrol and EX527 in DM and AD	3.54 ± 0.31^&^	3.89 ± 0.32^&^	273.55 ± 29.85^&^	315.36 ± 27.97^&^

*AchE activity was determined by Ellman’s colorimetric method and ChAT activity was determined by the radiochemical method. Data are expressed as mean ±SD. ^∗^*P* < 0.05 vs. normal control group. ^#^*P* < 0.05 vs. model group. ^&^*P* < 0.05 vs. resveratrol treatment group.*

### Cortical and Hippocampal Levels of Malondialdehyde and Glutathione and Superoxide Dismutase Activity

As reported in Table 2, a significant increase in cortical and hippocampal levels of MDA and concurrent significant decrease in SOD activity and GSH levels were observed in rats from the disease model group relative to healthy controls (*P* < 0.05). A significant decrease in cortical and hippocampal MDA levels and concurrent significant increase in SOD activity and GSH levels were observed (*P* < 0.05) in resveratrol-treated rats relative to model rats (*P* < 0.05, Table 2). Finally, a significant increase (*P* < 0.05) (Table 2) in cortical and hippocampal levels of MDA and concurrent significant decrease in SOD activity and GSH levels were observed (*P* < 0.05) (Table 2) in the resveratrol- and EX527-treated rats versus rats receiving resveratrol alone (*P* < 0.05) (Table 2), indicating the ability of the Sirt1 inhibitor EX527 to partially reverse the effects of resveratrol.

**TABLE 2:** MDA levels, SOD activity, and GSH levels in the cortex and hippocampus.

	MDA levels (nmol/mg protein)		SOD activity (U/mg protein)		GSH levels (nmol/mg protein)	
	Cortex	Hippocampus	Cortex	Hippocampus	Cortex	Hippocampus
Control	14.23 ± 1.3	10.24 ± 0.91	3.56 ± 0.32	4.57 ± 0.39	55.25 ± 5.20	102.97 ± 9.72
DM and AD	22.92 ± 0.25^∗^	18.47 ± 1.13^∗^	1.26 ± 0.11^∗^	2.25 ± 0.24^∗^	34.16 ± 3.31^∗^	74.86 ± 8.61^∗^
Resveratrol control	14.78 ± 1.16	9.98 ± 1.02	3.46 ± 0.31	4.72 ± 0.35	53.27 ± 5.25	104.62 ± 9.63
Resveratrol in DM and AD	17.04 ± 1.43^#^	13.16 ± 1.23^#^	2.28 ± 0.23^#^	3.78 ± 0.31^#^	46.27 ± 4.21^#^	91.92 ± 8.74^#^
Resveratrol and EX527 control	14.49 ± 1.21	10.39 ± 1.16	3.43 ± 0.37	4.65 ± 0.39	54.42 ± 4.93	105.37 ± 10.07
Resveratrol and EX527 in DM and AD	20.37 ± 1.86^&^	16.58 ± 1.45^&^	1.74 ± 0.15^&^	2.78 ± 0.26^&^	36.75 ± 4.12^&^	80.73 ± 9.27^&^

*Malondialdehyde levels were determined using thiobarbituric acid spectrophotometric colorimetry, SOD activity was determined using nitrite spectrophotometry, and GSH was determined spectrophotometrically using the DTNB-GSH reductase recycling method. Data are represented as mean ±SD. ^∗^*P* < 0.05 vs. normal control group. ^#^*P* < 0.05 vs. model group. ^&^*P* < 0.05 vs. resveratrol treatment group.*

### Cortical and Hippocampal Levels of IL-1β and IL-6

As shown in Table 3, a significant increase in cortical and hippocampal levels of IL-1β and IL-6 was observed in rats from the disease model group versus healthy controls (*P* < 0.05). Conversely, cortical and hippocampal levels of IL-1β and IL-16 were significantly decreased in resveratrol-treated rats compared to model rats (*P* < 0.05) (Table 3). However, a significant increase in cortical and hippocampal levels of IL-1β and IL-6 in rats treated with both resveratrol and EX527 was observed (*P* < 0.05) relative to rats receiving resveratrol alone (*P* < 0.05) (Table 3), indicating the ability of the Sirt1 inhibitor EX527 to partially reverse the effects of resveratrol.

**TABLE 3:** IL-1β and IL-6 levels in the cortex and hippocampus.

	IL-1β (U/mg protein)		IL-6 (U/mg protein)	
	Cortex	Hippocampus	Cortex	Hippocampus
Control	1.54 ± 0.146	1.683 ± 0.125	1.297 ± 0.136	1.426 ± 0.151
DM and AD	2.756 ± 0.157^∗^	2.946 ± 0.235^∗^	2.694 ± 0.217^∗^	2.975 ± 0.225^∗^
Resveratrol control	1.60 ± 0.147	1.749 ± 0.208	1.327 ± 0.178	1.512 ± 0.172
Resveratrol in DM and AD	2.13 ± 0.192^#^	2.34 ± 0.185^#^	1.963 ± 0.231^#^	2.13 ± 0.201^#^
Resveratrol and EX527 control	1.615 ± 0.135	1.712 ± 0.176	1.317 ± 0.91	1.482 ± 0.123
Resveratrol and EX527 in DM and AD	2.364 ± 0.181^&^	2.687 ± 0.197^&^	2.305 ± 0.123^&^	2.583 ± 0.215^&^

*IL-1β and IL-6 levels were determined by ELISA. Data are expressed as mean ±SD. ^∗^*P* < 0.05 vs. normal control group. ^#^*P* < 0.05 vs. model group. ^&^*P* < 0.05 vs. resveratrol treatment group.*

### Sirt1 Expression in the Brain

**FIGURE 4:**
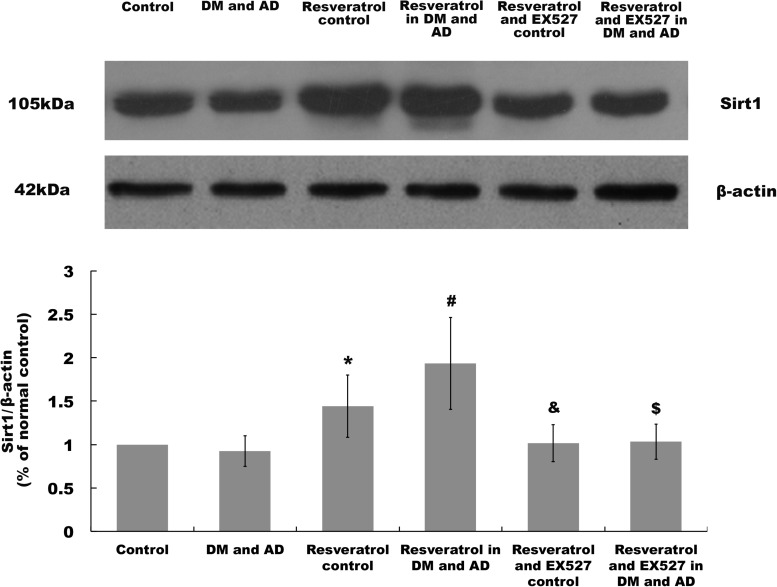
Sirt1 expression in the brain. Sirt1 expression in the brain was determined by Western blot. Three independent experiments were performed. Data are expressed as mean ±SD (^∗^*P* < 0.05, vs. control group; ^#^*P* < 0.05, vs. DM and AD group; ^&^*P* < 0.05, vs. resveratrol control group; ^$^*P* < 0.05, vs. resveratrol in DM and AD group).

Sirt1 expression in the brain was measured by Western blot analysis. As shown in Figure 4, a significant increase in expression of Sirt1 was observed in rats from the resveratrol control group and resveratrol treatment group versus healthy controls (*P* > 0.05) (Figure 4) and model rats (*P* > 0.05) (Figure 4), respectively. However, in the resveratrol and EX527 control rats as well as the resveratrol- and EX527-treated rats, all of the changes induced by resveratrol were reversed (*P* < 0.05) (Figure 4).

## Discussion

Traditionally, AD and DM have been considered two independent diseases. However, recent studies have shown that they share several pathophysiological mechanisms, including specific inflammatory signaling pathways (Bozluolcay et al., 2015), oxidative stress, and cell apoptosis. For example, in T2DM, impaired insulin signaling can result in the formation of Aβ plaques, mitochondrial dysfunction, inflammatory responses, and oxidative stress in peripheral tissues. The central nervous system exhibits all these pathological changes in AD, as well (I. Kim et al., 2010; Mittal and Katare, 2016). However, no therapeutic interventions are available that simultaneously address AD and DM.

Clinically, resveratrol exhibits numerous broad benefits that may help control many conditions, like DM (Shen et al., 2013; Szkudelski and Szkudelska, 2015) and AD (Vingtdeux et al., 2008; Li et al., 2012). For example, resveratrol can affect both glucose and lipid metabolism, and it was found to protect pancreatic β cells in a spontaneous T2DM model due to its as yet unexplained ability to induce activation of AMP-activated protein kinase and its downstream targets (Do et al., 2012). There is evidence that oxidative damage and subsequent deficits in cognition can be prevented via routine dosing of resveratrol as shown in another animal model of AD (Kim et al., 2007; Kumar et al., 2007). Resveratrol has also been found to inhibit Aβ secretion in various cell lines (Marambaud et al., 2005), prevent the formation and elongation of Aβ fibrils, and destabilize plaques (Mishra et al., 2009). A model of diabetes with AD in rats was developed for our study based on streptozotocin injection via the intraperitoneal route followed by hippocampal injection of Aβ1–40 to examine the effect of resveratrol. Our study suggests that resveratrol can protect the function of spatial sense and memory in the combined DM and AD rat model. In addition, examination of the cortex and hippocampus showed that resveratrol partially reversed the increase in AchE activity and the decrease in ChAT activity in the DM and AD rat model. These findings suggest that AchE and ChAT changes in the cortex and hippocampus may correlate with spatial sense and memory dysfunction in the rat model, and resveratrol may protect memory function in rats with concurrent diabetes and AD.

Among the final products of lipid peroxidation is MDA, which can be measured as an indicator of oxidative stress (Greilberger et al., 2008). SOD catalyzes the dismutation of the superoxide anion radical to either H_2_O_2_ or O_2_, regulating antioxidant defenses. GSH is noted for mitigating oxidative stress within cells by maintaining the redox state (Takahashi, 2012). Our results indicate that resveratrol treatment can partially reverse increased MDA levels and decreased SOD activity and GSH levels in the cortical and hippocampal brain regions of model rats. Thus, resveratrol exhibits significant antioxidant effects in the animal model of concurrent diabetes and AD.

One of the defining pathological changes responsible for sustaining the inflammatory response is cytokine production, and numerous studies have reported that cytokines are increased in the brains of both AD patients and diabetic patients (De Luigi et al., 2001; Devaraj et al., 2006). For example, IL-1β expression is increased sixfold in AD patients versus healthy controls of the same age (Griffin et al., 1989). IL-6 is also reportedly elevated in amyloid plaques found in the cortical and hippocampal tissue of AD patients. Devaraj et al. (2006) have reported that IL-6 and IL-1β levels were elevated in blood samples of type 1 diabetic patients versus samples from controls. Our results showed increases in IL-1β and IL-6 levels in model rats relative to normal rats, with resveratrol treatment partially reversing these changes. These findings suggest the existence of an inflammatory process in rats with concurrent diabetes and AD and indicate that resveratrol can interrupt the inflammatory cascade in the animal model of combined diabetes and AD.

Recently, many studies have suggested that Sirt1 has neuroprotective effects that slow the degeneration common to many neurological diseases, such as AD, Huntington’s disease, and Parkinson’s disease (Jiang et al., 2012; Lalla and Donmez, 2013; Herskovits and Guarente, 2014), and it is important for the regulation of many functions, including metabolism, stress tolerance, cell survival and aging, the inflammatory immune response, endothelial function, and circadian rhythm (Chung et al., 2010). Sirt1 modulates inflammatory reactions through deacetylating histones and critical transcription factors, such as activator protein 1 and nuclear factor kappa B (NF-κB), which block transcription of specific genes promoting inflammation (Xie et al., 2013). Olmos et al. (2013) found that Sirt1 supports vascular endothelial cells by regulating antioxidant genes via a FoxO3a/PGC-1α complex. Kobayashi et al. (2005) also found that Sirt1 impacts cellular aging and tolerance to stress by mediating NAD-dependent deacetylation of FOXO in a process triggered by oxidative signals. Studies have suggested that resveratrol is a Sirt1 activator (Pallas et al., 2009; Wu et al., 2011) and that Sirt1 is integral to the main neuroprotective mechanism of resveratrol. Moreover, the AMPK (Chiang et al., 2018; Guo and Zhang, 2018; Pineda-Ramirez et al., 2019), PI3K-AKT (Yin et al., 2017; Hui et al., 2018), and cAMP (Zhang et al., 2013) signaling pathways are also involved in resveratrol’s protective action in some disease models. In this study, we found that Sirt1 expression was significantly increased in the brains of rats from the resveratrol control group and resveratrol treatment group versus healthy controls and model rats, respectively, and that these changes were reversed by the Sirt1 inhibitor EX527. Our study demonstrates the partial reversal of resveratrol’s beneficial effects in a model of concurrent diabetes and AD in rats by coadministration of resveratrol with a Sirt1 inhibitor. Our findings suggest that resveratrol provides a protective effect in the animal model of combined diabetes and AD via activation of Sirt1 and its downstream targets. In addition, there may be additional signaling pathways, such as the AMPK, PI3K-AKT, and cAMP pathways, involved in resveratrol’s neuroprotection in the model of DM and AD, and further research is needed to confirm this.

## Conclusion

Resveratrol prevents neurodegeneration in a rat model of diabetes with concurrent AD by activating Sirt1 and its downstream targets to regulate the cholinergic system and control oxidative stress and the inflammatory response.

## Data Availability Statement

The raw data supporting the conclusion of this article will be made available by the authors, without undue reservation, to any qualified researcher.

## Ethics Statement

The animal study protocol was preapproved by the Life Science Research Ethics Committee of Zhengzhou University, and all procedures were conducted in accordance with the Guidance for the Care and Use of Laboratory Animals, formulated by the Ministry of Science and Technology of China.

## Author Contributions

XM, ZS, XH, and SL designed and/or performed the experiments. XM, ZS, XJ, and SC analyzed the data. ZS and XM wrote the manuscript. SC and JZ critically revised the manuscript. HL and JZ finally approved the manuscript.

## Conflict of Interest

The authors declare that the research was conducted in the absence of any commercial or financial relationships that could be construed as a potential conflict of interest.

## Abbreviations

Aβ: amyloid β-protein
AchE: acetylcholinesterase
AD: Alzheimer’s disease
BCA: bicinchoninic acid
ChAT: choline acetyltransferase
DM: diabetes mellitus
DMSO: dimethyl sulfoxide
DTNB: 5,5-dithiobis(2-nitrobenzoic acid)
ELISA: enzyme-linked immunosorbent assay
GSH: glutathione
MDA: malondialdehyde
MWM: Morris water maze
NBT: nitroblue tetrazolium
NF- κ B: nuclear factor kappa B
PVDF: polyvinylidene difluoride
SOD: superoxide dismutase
STZ: Streptozotocin
T2DM: type 2 diabetes mellitus.
